# Supplementary material for: Directed cortico-limbic dialogue in the human brain
Source: Nat Commun. 2026 Feb 2;17:2258. doi: 10.1038/s41467-026-68701-z (PMC12966484; doi:10.1038/s41467-026-68701-z)
Supplement: Supplementary file 2 — Reporting Summary [file 41467_2026_68701_MOESM2_ESM.pdf]

nature portfolio

Corresponding author(s): Maxime Baud

Last updated by author(s): Dec 8, 2025

Reporting Summary

Nature Portfolio wishes to improve the reproducibility of the work that we publish. This form provides structure for consistency and transparency in reporting. For further information on Nature Portfolio policies, see our Editorial Policies and the Editorial Policy Checklist.

Statistics

For all statistical analyses, confirm that the following items are present in the figure legend, table legend, main text, or Methods section.

n/a	Confirmed	
☐	☒	The exact sample size (*n*) for each experimental group/condition, given as a discrete number and unit of measurement
☐	☒	A statement on whether measurements were taken from distinct samples or whether the same sample was measured repeatedly
☐	☒	The statistical test(s) used AND whether they are one- or two-sided<br>*Only common tests should be described solely by name; describe more complex techniques in the Methods section.*
☐	☒	A description of all covariates tested
☐	☒	A description of any assumptions or corrections, such as tests of normality and adjustment for multiple comparisons
☐	☒	A full description of the statistical parameters including central tendency (e.g. means) or other basic estimates (e.g. regression coefficient) AND variation (e.g. standard deviation) or associated estimates of uncertainty (e.g. confidence intervals)
☐	☒	For null hypothesis testing, the test statistic (e.g. *F*, *t*, *r*) with confidence intervals, effect sizes, degrees of freedom and *P* value noted<br>*Give P values as exact values whenever suitable.*
☒	☐	For Bayesian analysis, information on the choice of priors and Markov chain Monte Carlo settings
☐	☒	For hierarchical and complex designs, identification of the appropriate level for tests and full reporting of outcomes
☐	☒	Estimates of effect sizes (e.g. Cohen's *d*, Pearson's *r*), indicating how they were calculated

Our web collection on statistics for biologists contains articles on many of the points above.

Software and code

Policy information about availability of computer code

Data collection	Data was collected with a Quantum® LTM Amplifier (Natus, Middleton, Wisconsin, USA).
Data analysis	All data was analyzed using custom code that is available at this link: https://github.com/neuro-elab/EvM_Connectivity/releases/tag/Connecto_v0.3. (Visualization) https://github.com/neuro-elab/EvM_Directed-cortico-limbic-dialog.git (Data and Analysis Scripts)

For manuscripts utilizing custom algorithms or software that are central to the research but not yet described in published literature, software must be made available to editors and reviewers. We strongly encourage code deposition in a community repository (e.g. GitHub). See the Nature Portfolio guidelines for submitting code & software for further information.

Data

Policy information about availability of data

All manuscripts must include a data availability statement. This statement should provide the following information, where applicable:

- Accession codes, unique identifiers, or web links for publicly available datasets
- A description of any restrictions on data availability
- For clinical datasets or third party data, please ensure that the statement adheres to our policy

All measurement data is available for download and visualization in the accompanying graphical user interface at https://github.com/neuro-elab/EvM_Connectivity/releases/tag/Connecto_v0.3.

Research involving human participants, their data, or biological material

Policy information about studies with human participants or human data. See also policy information about sex, gender (identity/presentation), and sexual orientation and race, ethnicity and racism.

Reporting on sex and gender	Sex and/or gender was not considered in the study design, nor inclusion criteria. Low sample size prevented sex- or gender-based analyses.
Reporting on race, ethnicity, or other socially relevant groupings	There no socially constructed categorization variable used in the study.
Population characteristics	See above.
Recruitment	Patients were recruited among patients with epilepsy undergoing intracranial EEG for clinical reasons.
Ethics oversight	Kanton Bern Ethik Kommission

Note that full information on the approval of the study protocol must also be provided in the manuscript.

Field-specific reporting

Please select the one below that is the best fit for your research. If you are not sure, read the appropriate sections before making your selection.

☒ Life sciences ☐ Behavioural & social sciences ☐ Ecological, evolutionary & environmental sciences

For a reference copy of the document with all sections, see nature.com/documents/nr-reporting-summary-flat.pdf

Life sciences study design

All studies must disclose on these points even when the disclosure is negative.

Sample size	Each participant has a unique brain coverage and we needed to pool enough participants to have coverage of all investigated brain regions. 15 participants were sufficient for this. Undoubtedly, future studies with more participants will enable refinement of the brain parcellation proposed here.
Data exclusions	Two participants had insufficient sleep data and had to be excluded from the final analysis, but could be included in the analyses of connectivity during wake.
Replication	Our measurements of signaling probability between two given brain regions was largely reproducible across participants. In some cases, larger variability was observed, likely because the parcellation of brain regions needs to be further refined.
Randomization	There was no randomization, nor group comparisons. Comparison of vigilance stages was done in a longitudinal design, in which each participant served as its own control.
Blinding	For the final analysis, vigilance stages were visually scored by two independent scorers who were blinded to the results of the other scorer.

Reporting for specific materials, systems and methods

We require information from authors about some types of materials, experimental systems and methods used in many studies. Here, indicate whether each material, system or method listed is relevant to your study. If you are not sure if a list item applies to your research, read the appropriate section before selecting a response.

Materials & experimental systems

n/a	Involved in the study	
☒	☐	Antibodies
☒	☐	Eukaryotic cell lines
☒	☐	Palaeontology and archaeology
☒	☐	Animals and other organisms
☐	☒	Clinical data
☒	☐	Dual use research of concern
☒	☐	Plants

Methods

n/a	Involved in the study	
☒	☐	ChIP-seq
☒	☐	Flow cytometry
☐	☒	MRI-based neuroimaging

Clinical data

Policy information about clinical studies

All manuscripts should comply with the ICMJE guidelines for publication of clinical research and a completed CONSORT checklist must be included with all submissions.

Clinical trial registration	KEK Bern 2018-01387
Study protocol	Not a trial. Ordinance for research with human.
Data collection	For all other analyses, data were collected between 2019-2024 from 15 adults (median [range] age 33 [19 to 63]) with pharmacoresistant epilepsy undergoing invasive presurgical evaluation at Inselspital Bern, Switzerland who consented to participate in research.
Outcomes	Primary outcome: signaling probability and directionality. Secondary outcome: change in signaling probability and directionality over the sleep-wake cycle.

Plants

Seed stocks	There is a problem with the form, when selecting N/A for plants, this section opens. There were no plants involved in our study.
Novel plant genotypes	There is a problem with the form, when selecting N/A for plants, this section opens. There were no plants involved in our study.
Authentication	There is a problem with the form, when selecting N/A for plants, this section opens. There were no plants involved in our study.

Magnetic resonance imaging

Experimental design

Design type	Structural MRI
Design specifications	Structural MRI for electrode localization
Behavioral performance measures	N/A.

Acquisition

Imaging type(s)	Structural
Field strength	3T
Sequence & imaging parameters	MPRAGE sequence
Area of acquisition	Whole brain scan.

Diffusion MRI ☐ Used ☒ Not used

Preprocessing

Preprocessing software	Advanced Normalization Tools. Statistical Parametric Mapping (SPM)
Normalization	Participant brain mapped to the MNI brain template.
Normalization template	MNI template
Noise and artifact removal	Electrode location
Volume censoring	N/A

Statistical modeling & inference

Model type and settings	N/A, structural imaging
Effect(s) tested	N/A, structural imaging

Specify type of analysis: [x] Whole brain [ ] ROI-based [ ] Both

Statistic type for inference (See Eklund et al. 2016): N/A, structural imaging

Correction: N/A, structural imaging

Models & analysis

n/a	Involved in the study
[ ]	[x] Functional and/or effective connectivity
[ ]	[x] Graph analysis
[ ]	[x] Multivariate modeling or predictive analysis

Functional and/or effective connectivity: We measured effective connectivity from evoked potentials, not MRI

Graph analysis: We analyzed individual connections (links) but did not derive more general graph metrics, due to concerns with out under-sampling of the brain.

Multivariate modeling and predictive analysis: Mixed effects linear models.
